# Changes to Refugee Mental Health During and After a Cross-Sector PTSD Intervention: A Qualitative Longitudinal Study About the Influence of Social Support, Life Events, and Agency

**DOI:** 10.1007/s11013-025-09944-1

**Published:** 2025-10-05

**Authors:** Henriette Laugesen Attardo, Maja Bruhn, Morten Skovdal, Jessica Carlsson, Åsa Audulv

**Affiliations:** 1 Section of Clinical Medicine, University of Copenhagen, Copenhagen, Denmark; 2 Competence Centre for Transcultural Psychiatry, Mental Health Centre Ballerup, Copenhagen University Hospital – Mental Health Services CPH, Copenhagen, Denmark; 3 Department of Nursing, Umeå University, Umeå, Sweden; 4 Section of Health Service Research, University of Copenhagen, Copenhagen, Denmark; 5 Centre for Culture and the Mind, University of Copenhagen, Copenhagen, Denmark

**Keywords:** Refugee, Post-traumatic stress disorder (PTSD), Mental health, Cross-sector collaboration, Qualitative Longitudinal Research

## Abstract

Cross-sector interventions are increasingly suggested in care for trauma-affected refugees, but knowledge about how they influence mental health over time remains sparse. Using a qualitative longitudinal design, we explored patterns of mental health change and aspects contributing to change among refugees participating in a cross-sector intervention addressing post-migration stressors alongside treatment for post-traumatic stress disorder. Interviews were conducted with participants at four time points during the intervention and the year after. A pattern-oriented analysis helped identify three patterns of mental health change, in which participants experienced either consistent improvements, decline without the support of the intervention, or persistently poor mental health despite changes to post-migration stressors. The patterns differed in how refugees (a) perceived benefiting from the intervention, (b) desired or benefited from social interactions and support, (c) encountered challenging life events, and (d) explained their expectations and agency. The intervention supported improved mental health in two patterns; however, lasting improvement beyond the intervention period was identified in only one pattern. We discuss adaptations and alternative interventions. Findings support a personalized and cross-sectoral approach to mental health treatment to better support the individual needs of refugee patients.

**Supplementary Information:**

The online version contains supplementary material available at 10.1007/s11013-025-09944-1.

## Introduction

Over the last decade, the number of refugees worldwide has more than tripled (United Nations High Commissioner for Refugees, 2024). Around 30% of refugees and asylum-seekers struggle with post-traumatic stress disorder (PTSD) and depression (Blackmore et al., 2020). According to Handiso et al. (2023), both PTSD and depression appear to persist for years after resettlement, indicating that post-migration stressors, in addition to emotional distress due to traumatic experiences, challenge mental health. For refugees who are long-settled, a clear association between post-migration stressors and mental disorders or lower well-being exists (Bogic et al., 2015).

Consequently, there is a need for mental health interventions that can simultaneously address emotional distress due to traumatic experiences *and* post-migration stressors. Suggested approaches include cross-sector interventions addressing coordination between existing services and multilevel programs targeting several stressors or multiple groups of recipients (Miller & Rasmussen, 2024). Research on the US Refugee and Immigrant Well-being Project shows that a partnership involving university students and community-based actors can reduce emotional distress and increase language proficiency and social support in newly arrived refugee families by focusing on practical assistance and mobilizing community resources (Goodkind et al., 2020). Studies of the Problem Management Plus intervention, developed to manage mental health problems and practical stressors in non-clinical populations, indicate that addressing both aspects in one intervention is favorable, even though the intervention’s effect on PTSD symptoms is small (Schäfer et al., 2023). While such interventions are promising, it has also been suggested that specialized clinical mental healthcare is likely to be needed for refugees with more complex care needs who do not benefit from lay-delivered or non-specialized interventions (de Graaff et al., 2024).

Miller et al. (2021) have argued that specialized clinical mental healthcare for refugees focuses predominantly on treating psychopathology without targeting distress caused by social determinants. Furthermore, a qualitative meta-synthesis by Khairat and colleagues (2023) emphasizes that engagement in specialized psychotherapy is challenging for refugees struggling with post-migration stressors. Refugees thus request that practical assistance to manage stressors be added to trauma-focused mental health treatment (Duden et al., 2020). Notably, a study of immigrant torture survivors resettled in the US demonstrated that combined psychotherapy and case management targeting post-migration stressors was more favorable than case management only (Reed et al., 2023). A case study from the United Kingdom also reports positive feedback on a multidisciplinary care approach where professional stakeholders across sectors attend virtual team meetings to develop integrated care plans for refugees with complex health and social service needs (Harris et al., 2024). These findings support that specialized mental healthcare for refugees needs adaptation, but research about how to address post-migration stressors in specialized clinical care contexts is currently limited. Addressing this gap is crucial to advancing specialized mental healthcare and bringing it up to par with the social determinants perspective adopted by community-based interventions for refugees.

We present findings from a novel cross-sector PTSD intervention in a specialized care setting that integrated management of post-migration stressors into standard multidisciplinary PTSD treatment encompassing psychoeducation, pharmacotherapy, and psychotherapy. This integrated approach reflects person-centeredness with coordination of services across sectors tailored to patients' needs, as proposed by Miller et al. (2024). We adopted a dual continuum understanding of mental health with psychopathology on the negative side and well-being dimensions on the positive side, encompassing flourishing, sense of agency, life satisfaction, and feelings of interconnectivity to people and context (Joshanloo & Weijers, 2019; Magalhães, 2024; World Health Organization, 2022). We thereby acknowledge that mental health is not solely an individual trait, but is profoundly shaped by socio-cultural and environmental factors. With this understanding of mental health, the study aimed to (a) qualitatively explore how refugees participating in the cross-sector PTSD intervention experienced changes in their mental health and (b) understand the aspects that contributed to different patterns of mental health change during and after the PTSD intervention.

## Materials and Methods

### Study Design and Setting

This Qualitative Longitudinal Research (QLR) study was nested within a randomized controlled trial testing the effectiveness of the cross-sector PTSD intervention (Bruhn et al., 2022). It took place at an outpatient PTSD clinic in Denmark. The current QLR study was underpinned by social constructivism, acknowledging multiple realities and that knowledge is shaped by interactions (Guba & Lincoln, 1994). QLR focuses on exploring changes over time and the mechanisms of change (Audulv et al., 2023). Therefore, QLR allowed an in-depth exploration of changes in mental health experienced by refugees over time, but also an exploration of what happened in their lives between data collection time points and how it related to their mental health. The intervention aimed to provide multidisciplinary PTSD treatment and coordinate services across sectors to manage post-migration stressors, thereby improving mental health. Multidisciplinary treatment encompassed psychoeducation, pharmacological treatment, and psychotherapy to enhance functioning and reduce symptoms (Bruhn et al., 2022). Many patients referred to the PTSD clinic had been unemployed long-term. Therefore, the intervention also aimed to assist in and expedite the work capacity assessment. In Denmark, this assessment is necessary when the ability to work is impaired. During the assessment process, activities are scheduled to find appropriate work, including individually tailored work with reduced hours or specific tasks. The process can lead to disability pension if all options to strengthen the capacity to work have been explored and documented, and the impairment is deemed permanent.

The intervention rationale was that managing post-migration stressors, including the work capacity assessment, could reduce perceived stress and thus advance PTSD treatment outcomes by making it easier to engage in multidisciplinary treatment. The results of the RCT are pending. With the present QLR, we provide insight into the factors within and outside the PTSD intervention that influence mental health over time. Consequently, as argued by other scholars, the findings can strengthen and explain RCT results, increase credibility, point to active ingredients influencing mental health improvements or non-improvements, and thus guide nuanced decision-making about managing post-migration stressors as part of specialized mental healthcare (Sørensen et al., 2023).

Collaboration and coordination were achieved by arranging three cross-sector network meetings during the 12-month specialized PTSD treatment as usual. Refugees themselves, their mental health professionals (physicians, psychologists, and mental health social workers employed at the PTSD clinic), their employment case workers from municipal employment services, and sometimes other relevant stakeholders (e.g., mentors or case workers from child and family services) participated in the network meetings (Bruhn et al., 2022). A preparatory session with the refugee participants was held one or two weeks before each network meeting to formulate an agenda. Between network meetings, the mental health professionals supported refugees in coping with post-migration stressors and discussed the content of shared action plans.

Three of the authors took part in intervention development, two authors are mental health clinicians, one is a social psychologist, and none of the authors are refugees themselves.

### Participants

Refugee participants were recruited for this QLR study through their mental health social worker after randomization to the intervention group. All 24 participants recruited were diagnosed with PTSD and affiliated with municipal employment services. A purposive sampling technique (Patton, 2015) was used to recruit participants with selected varying life circumstances, including the type of social assistance received, living situation, and country of origin. One participant dropped out of the intervention, and one participant could not participate in all interviews due to an increasing severity of mental health symptoms. The remaining 22 participants completed the intervention and participated in end-of-intervention interviews. Of these, 18 were available for follow-up interviews one year after the intervention and were therefore included in this study. The remaining four either did not respond to an invitation for the follow-up interview or expressed feeling too sick or busy to participate. On average, the 18 participants were 50 (38-61) years old at the first interview, had lived in Denmark for 20 years (range 3-35) prior to the network meetings, and their countries of origin were Afghanistan, Iraq, Iran, Lebanon, Somalia, Syria, and Turkey. Nine participants were women, and nine were men.

### Ethical Considerations

The trial, including the qualitative data collection for this study, was assessed by the Regional Ethics Committee in Denmark (H-19067136), which waived the need for ethical approval. Permission was obtained from the Danish Data Protection Agency (P-2019-327). All participants signed consent forms that were available in Danish, Arabic, and Farsi. Due to the longitudinal nature of the study, consent was discussed at each time point. No participants withdrew consent.

### Data Collection and Analysis

**Fig. 1:**
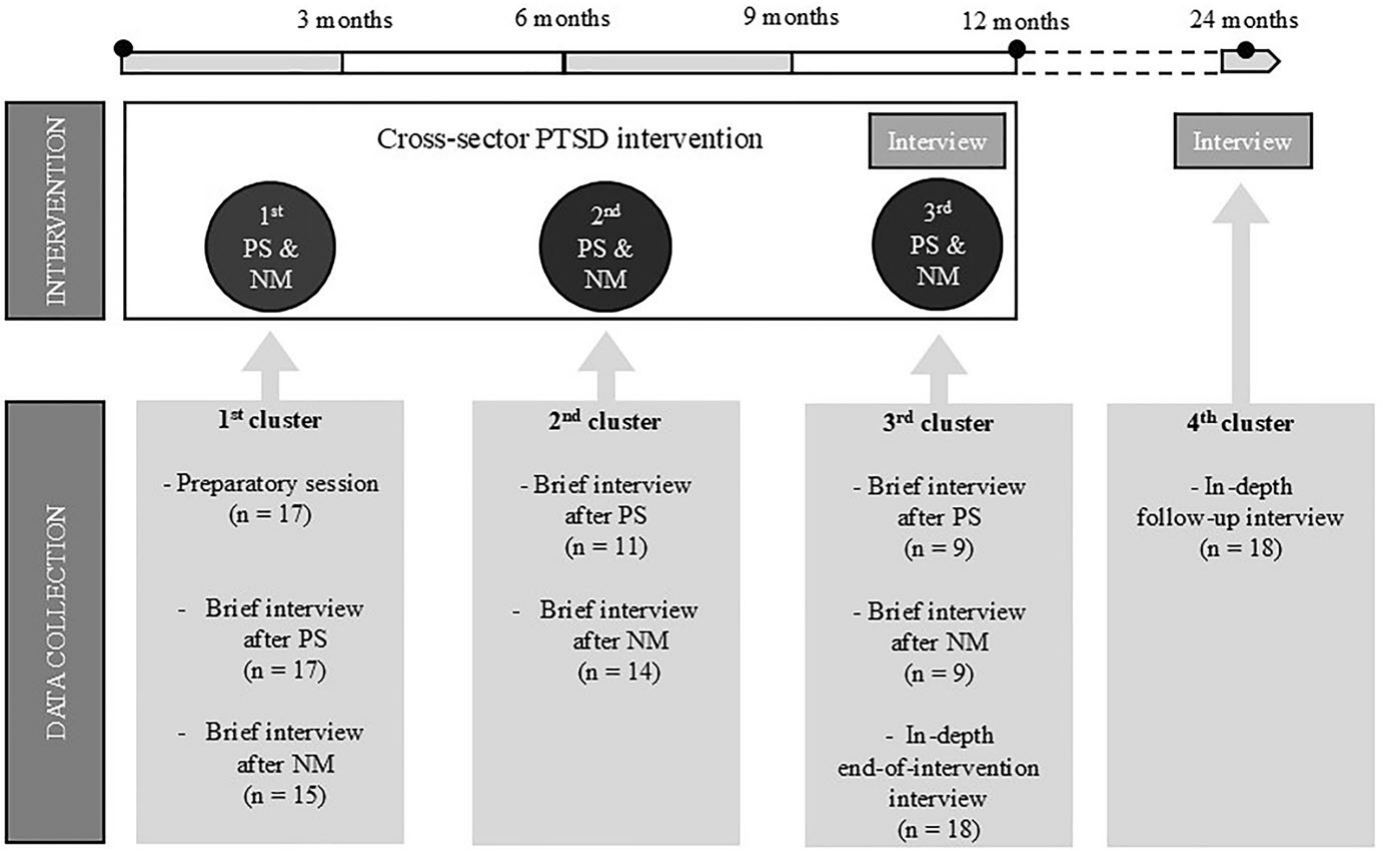
Data collection process during and after the cross-sector PTSD intervention at four clustered time points. *PS* preparatory session, *NM* network meeting

Data were collected at four clustered time points for each participant, depicted in Figure 1. Data were predominantly collected through interviews, which were informed by preceding participant observations. For time point one, data collected during observations, including recordings of the first preparatory session, were analyzed because the participants thoroughly described their life circumstances and challenges during these sessions. For all other time points, only interview transcripts were analyzed.

Clustered time point one encompassed observation/audio recording of the first preparatory session, and brief interviews conducted after the first preparatory session and the first network meeting. These interviews briefly explored life circumstances, mental health, and expectations towards network meetings. Clustered time point two consisted of brief interviews after the second preparatory session and the second network meeting. Time point three included brief interviews conducted after the third preparatory session and the third network meeting, as well as an in-depth end-of-intervention interview, which had an average length of 45 minutes (range 28–55 min). Time point four covered an in-depth follow-up interview, scheduled approximately one year after the end-of-intervention interview, which took an average of 45 minutes (range 17–56 min). The in-depth interviews explored the participants’ thoughts about the future, their mental health, everyday life and social circumstances during and after the PTSD intervention. The interview guides are available in a supplementary file. Most interviews were conducted in person (*n* = 101), while a few (*n* = 10) were conducted over the phone, as preferred by the participants. Trained interpreters were used for 14 of the participants. The first author, HLA, conducted all the data collection between June 2021 and June 2024 and had previous experience in undertaking interviews.

Data were analyzed using a Pattern-Oriented Longitudinal Analysis (POLA) (Kneck & Audulv, 2019), in which multiple data collection time points are used to identify different patterns of change over time across study participants, thereby identifying variations in changing patterns. Using a two-stage process, HLA first coded data to identify descriptions of mental health and aspects that the participants linked to their mental health. These aspects were *hope, worries*, *identity and autonomy, PTSD intervention*, *unemployment process, practical support,* and *social life*. Coding strategies were discussed in research group meetings. For the second stage, data were arranged in a matrix to visualize change over time for each individual (Kneck & Audulv, 2019). Matrices included coded data extracts of mental health and related aspects at each clustered time point. Table 1 displays an example of two aspects in Khalid’s individual matrix.

**Table 1:** Matrix example including two aspects.

Aspect	1^st^ clustered time point	2^nd^ clustered time point	3^rd^ clustered time point	4^th^ clustered time point
PTSD intervention	Khalid has the impression that professionals are actively trying to help, which makes his mood better.	Khalid still feels that professionals are trying to help. It makes him happy to see that people are trying.	Khalid says the medicine has improved his sleep a bit, but psychotherapy did not help. Khalid applauds the professionals for making efforts to address stressors during network meetings. He believes that network meetings have been beneficial.	Khalid believes the network meetings helped him receive disability pension and attain a new apartment.
Hope	Khalid has problems that have persisted for a long time. He believes that lasting solutions cannot be found. He feels caught in bureaucracy and thrown around like a ball.	Khalid describes having an abundance of problems and not enough strength to deal with them all. Trying to make changes is a waste of time.	Khalid has limited hopes for a better future. He thinks he has too many problems and life will never be great.	Khalid feels that he persistently encounters new challenges. There is never a reprieve.

The second stage began with a research group meeting, where the matrices were sorted according to similarities and differences per the participants’ descriptions of mental health changes. Three patterns were identified in the process. HLA then further examined the individual matrices. Differences both within and between patterns were scrutinized in research group meetings. This led to additional revisions before the final pattern characteristics were determined.

## Results

Three patterns of mental health change were identified. Participants sharing pattern 1 (*n* = 6) described improved mental health during the PTSD intervention and after. The participants who followed this pattern described having an inherent ability to create change, and they started to seek social interactions during the intervention period. Participants sharing pattern 2 (*n* = 5) described improved mental health during the intervention. However, after the intervention ended, they described a decline due to challenging life events, a lack of support to deal with challenges, and a desire for more social interactions. Participants sharing pattern 3 (*n* = 7) described smaller fluctuations during and after the intervention, but overall, their mental health was persistently poor, and they also had severe somatic health challenges. They preferred a calm life in solitude. Table 2 provides a comparative overview of pattern characteristics.

**Table 2:** Comparative table of characteristics across patterns during the data collection period.

Pattern description	Pattern 1 (*n* = 6) Experience continuous improvements and demonstrate agency during intervention and beyond.	Pattern 2 (*n* = 5) Experience improvements through intervention support, but setbacks without support.	Pattern 3 (*n* = 7) Experience no improvements but have access to support and accept status quo.
Demographic characteristics	• 5 women, 1 man • Average age 49 • 1 arrived after 2014^a^ • 5 needed interpreters^b^ • Live mostly with family	• 2 women, 3 men • Average age 51 • 2 arrived after 2014^a^ • 4 needed interpreters^b^ • Live mostly with family	• 2 women, 5 men • Average age 50 • 1 arrived after 2014^a^ • 5 needed interpreters^b^ • Live mostly alone
Expectations and agency	• Recognize ability to cope and reach out for help when needed. • Encounter positive experiences and find hope. • Desire and achieve employment or daily activities.	• Desire partnerships and feel lost without the assistance and support of others. • Lose hope at the end of or after the intervention because things do not go as planned. • Desire employment but do not achieve it.	• Let the professionals take the lead to get through procedural motions quicker. • Accept the situation and lack hope for the future. • Find employment unrealistic.
Social interactions and support	• Seek and find social interactions meaningful. • Describe no unmet support needs at follow-up.	• Like but do not seek social interaction. • Describe several unmet support needs at follow-up.	• Prefer solitude. • Describe no unmet support needs at follow-up.
Life events during the study period	• Encounter few challenging events. • Encounter no health-related events.	• Encounter many challenging events. • Encounter combinations of health-related events and non-health-related events.	• Encounter one or two challenging events. • Encounter primarily health-related events.

^a^Denmark experienced an increased influx of refugees after 2014. Changes to asylum and permit procedures were made in 2015

^b^Interpreters were needed during multidisciplinary treatment, network meetings, and interviews

The demographic characteristics were quite similar across the patterns, for example, in terms of age, years of living in Denmark, and Danish language proficiency. However, the gender composition differed across the patterns, with participants in pattern 1 being predominantly women and those in pattern 3 mainly being men.

### Pattern 1: Experience Continuous Improvements and Demonstrate Agency During Intervention and Beyond

Six participants described lasting improvements to mental health, while their somatic conditions (such as fibromyalgia or arthritis) were often chronic. Several described living with daily pain at all time points, but also had a desire to engage in daily activities such as working or visiting community centers. When starting the PTSD intervention, they described mental health struggles, then gradually benefited from medication and psychotherapy. They found the intervention brought relief, and they gained new knowledge about their mental disorder and why it developed. As the intervention concluded, the participants reported improvements in their mental health compared to how they felt at the start of the intervention. For example, they reported being less prone to crying, less irritated, more energized, more interested in socializing, and their sleep had improved. Hence, the participants benefited from the multidisciplinary treatment addressing emotional distress. Amal explained her change during the intervention period:

A: When I came here [to the clinic], I felt so stressed, and you could not speak with me. I cried if you spoke with me. I could not sleep. [I was] like a broken glass, and you start to put it back together again. This is what happened to me here. I feel like this. Because when I first came here [to the clinic], I cried a lot. But after, I felt better, that I did not need to cry anymore. Right now, I feel strong. They gave me medicine that has helped me sleep again and many [other types of] treatment, like not medicine, but something to do [such as coping strategies].

(Amal T3)

In addition to the multidisciplinary treatment, the participants described how the network meetings helped them because their support needs were addressed, and they felt helped and listened to by the professionals. All participants agreed that the main benefit of the network meetings was that the mental health professionals could explain their mental health challenges to the employment case workers:

R: It was important to me that the [employment service in the] municipality knows how I am doing. That was the most important thing for me [during network meetings and] to collaborate around me, [having] the different departments collaborate around me.

I: Do you think that has resulted in greater collaboration around you?

R: Yes, I could feel it benefiting me (…). The [employment service] needed to know how much [daily activity] I can manage, how much I cannot manage, what treatment I receive, and what medication I receive. It was important to me that they knew that [at the municipal employment service].

(Ralia T3)

Furthermore, the participants described how the network meetings contributed to progression in their work capacity assessment, and four of the six participants received final assessment decisions at some point during the study period. It seems the intervention's attention to managing post-migration stressors benefited the participants and supported progression alongside multidisciplinary PTSD treatment. By time point four, no participants expressed unmet support needs, and they described their mental health as maintained or improved. The participants described using coping strategies learned during the intervention to sustain their mental health, such as diversion and anger management.

The participants shared the perception that they could make changes in their lives. Some participants recognized that they had change agency at the beginning of the intervention. Others described that it developed over time. For example, Wafa described being passive in an early interview, whereas at time point three she explained taking initiatives to change things:

W: I have started to think that if there is something that is not as it should be. Then I start thinking about what I can do about it. If I cannot do anything about it, then I get busy or occupy myself with something else. That way, I do not focus on and think about it.

(Wafa T3)

**Fig. 2:**
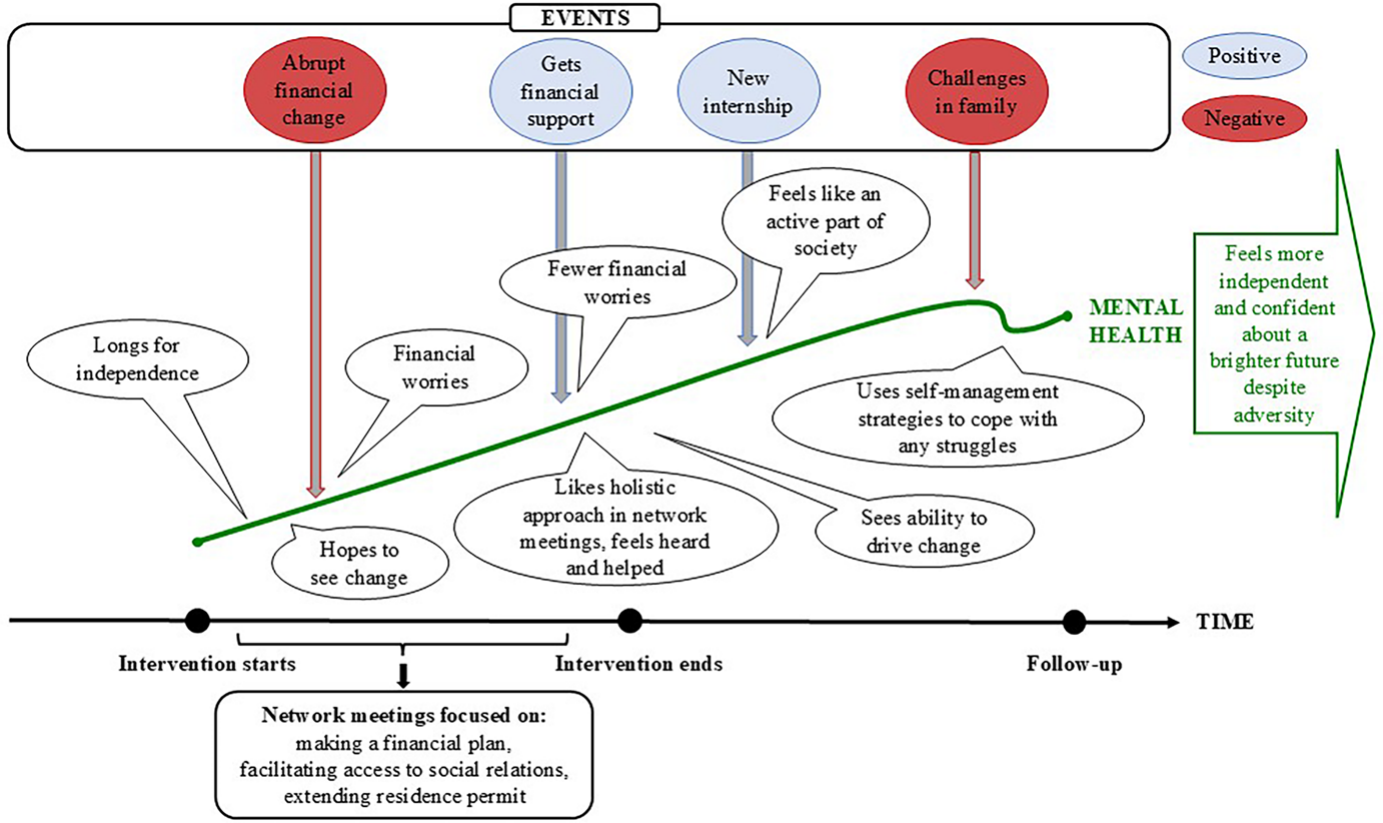
Example of mental health journey through time for pattern 1.

During the intervention period, the participants described limited engagement in social interaction outside their immediate family, with whom they often lived. After the intervention they engaged more in social interactions through voluntary work and community centers, or they had started to entertain the idea of being more social: *“I have also become better at socializing. I have great colleagues [at the internship] (…). At first it was difficult [to socialize], but now I know people”* (Basel T4). Figure 2 provides an example of the mental health journey for participants sharing pattern 1.

### Pattern 2: Experience Improvements Through Intervention Support but Setbacks Without Support

Five participants shared mental health patterns with improvements and subsequent setbacks. When starting the intervention, the participants described poor mental health influenced by a combination of somatic problems, severe pain, and past trauma. They felt forgetful, jumpy, had difficulties concentrating, and experienced poor sleep. Despite this, four of the five participants said they wanted to work. The fifth expressed a desire to work but did not deem it realistic. The participants described how their somatic health problems and long-term pain persisted during the intervention, but their mental health improved:

I: Can you explain the change you have experienced since starting treatment?

A: It is a huge difference. When I came here in the beginning, I could feel it pinching like this [she curls her body completely inwards, places a clenched hand on her chest to indicate that all the traumatic experiences she carried with her caused a restriction that put a strain on her body].

I: And now?

A: Now I feel better, thank God [she sits up straight again and smiles].

(Anab, T3)

The participants described feeling helped by the intervention because they could share their burdens with professionals, receive advice, and learn coping strategies. Thus, they benefited from the multidisciplinary PTSD treatment. They explained that the network meetings created a sense of hope for progress due to the additional access to support and help. Despite this hope, the intervention’s focus on addressing post-migration stressors did not benefit all participants sharing pattern 2. While two participants expressed satisfaction with the progress made through network meetings, the other three participants experienced a lack of progress by the end of the intervention, which disappointed them. They noted that network meetings seemed like a good idea on paper, but in reality, the meetings were all talk and no real action:

D: We have talked about all of the things that are important to me [during the network meetings], but nothing happened. Thus, everything that I had wanted [resolved] did not change. It [the network meetings] was more of a cozy chat.

(Deqa, T3)

After the intervention, the participants described losing much of the mental health improvement gained during the intervention. The participants were not employed at time point four, and their previous hopefulness, sparked by the intervention, had diminished. Instead, they expressed concerns about their futures, anticipating negative outcomes, and felt frustrated with their situations.

This mental health decline seemed linked to their encounters with new challenging life events and having unmet support needs after the intervention. The participants described suddenly facing new or worsened somatic health problems, combined with either family issues or negative experiences related to employment service activities. Through the intervention, four of the five participants got access to support and companionship, such as voluntary friends or mentors. However, when the intervention ended, they lost this support and were left to deal with post-migration stressors themselves. Omar described his experience in the following way:

O: I was really sad when I had to stop [with the treatment and cross-sectoral support provided during the intervention].

I: How come you were sad when you had to stop?

O: Because I felt that I was abandoned in the middle of the road (…). I could feel that if I had continued [receiving the intervention] a little longer, I could have gotten the complete help that I needed, but it was an abrupt stop in the middle of it all.

(Omar, T4)

**Fig. 3:**
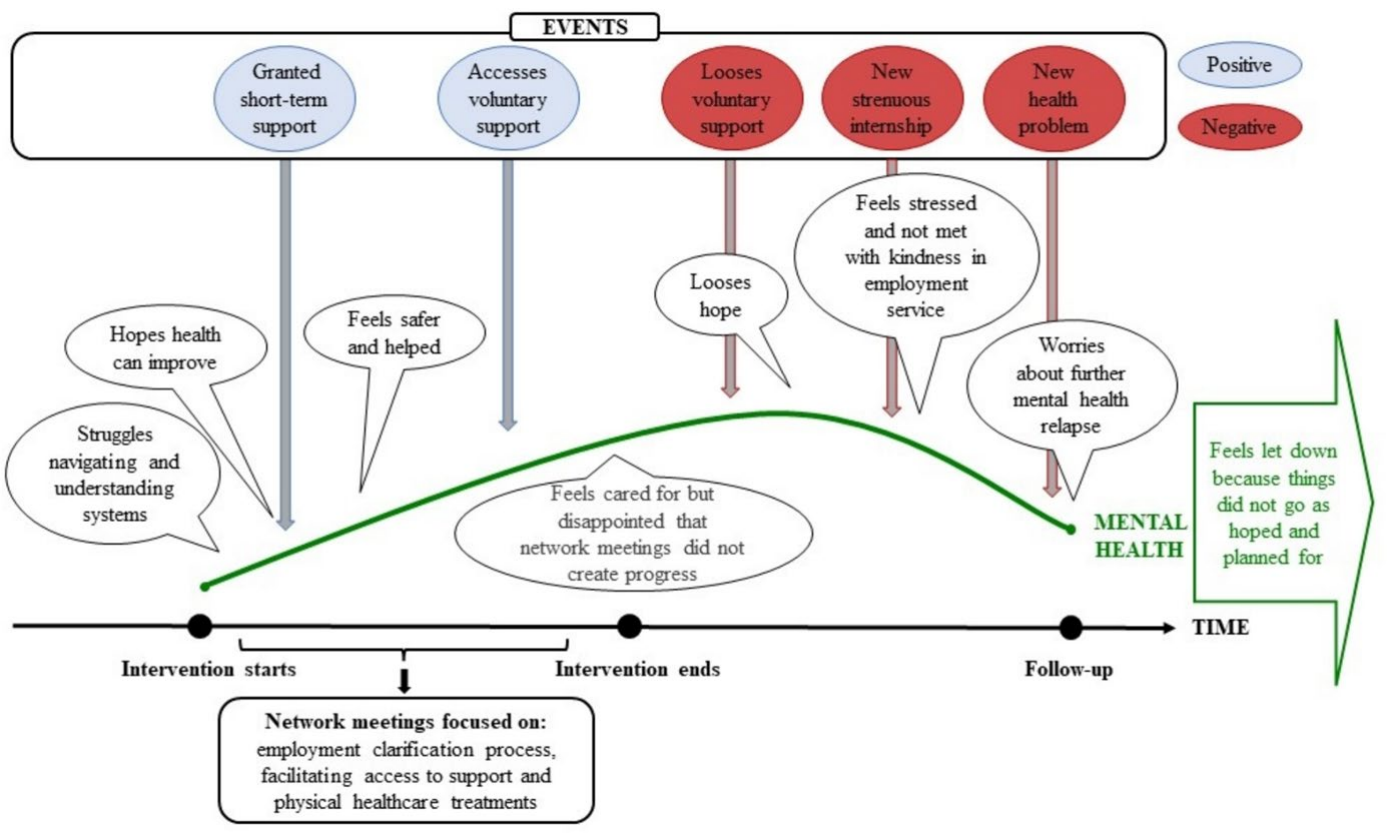
Example of mental health journey through time for pattern 2.

As exemplified in this quote, the participants felt lost and were left to deal with their post-migration stressors without the support that the intervention had facilitated. Notably, the intervention only facilitated access to short-term and unstable support, rather than long-term and stable support, due to the voluntary nature of companionships and fixed 13-week mentorships. Figure 3 provides an example of the mental health journey for participants sharing pattern 2.

### Pattern 3: Experience no Improvements but has Access to Support and Accepts Status Quo

Seven participants experienced persistently poor mental health with smaller fluctuations. They explained how all their challenges made them feel caught in a vicious circle. The participants stated that the professionals were nice, but the intervention did not contribute to positive mental health change. The participants described being too sick, beyond help, and attributed their chronically poor mental health to previous traumatic experiences, multimorbid somatic health issues, and lasting severe pain. They described being depressed, having trouble sleeping, and experiencing anxiety, which in combination made social interactions challenging. In the extracts below, Mohammad described the many challenges that put a strain on his life, which overshadowed the positive change regarding his accommodation:

M: Problems keep coming up all the time. Not being able to afford things, and depression, and rent [for the apartment], and then there are the other problems. So how am I supposed to be part of society? And plus, I am in a bad mood and cannot stand [being] with people.

(Mohammad, T1)

M: It [mental health] has gotten even worse. And plus, I got something called otoliths two months ago.

(Mohammad, T2)

M: P: Physically I am [doing] very bad. I have all sorts of diseases. The sugar [diabetes type 2] started, and vitamin D [deficiency], [high] cholesterol, and I discovered that in my back I still have the same problem [a herniated disc]. And in the last two days it has been pressing on my heart again. (…). And the sleep problem continues. [I am in a] bad mood all the time and I cannot be anywhere. It is still going on like that. But luckily, I got my [new] apartment.

(Mohammad, T3)

The participants explained how employment services made them feel caught and stuck. All but one participant had been affiliated with employment services for over five years, and all desired to be free from appointments and obligations. They wanted an official decision about their work capacity assessment to be made, and did not think that employment was realistic. The participants expressed how their primary goal was to convince the employment case workers of the legitimacy of their health struggles and inability to work. The participants thus needed the mental health professionals to validate their condition during network meetings, as explained by Ahmad:

A: I think [the network meetings] have been useful, otherwise we would not be able to present my condition in detail [to the employment case worker].

I: What has been the best thing about the network meetings?

A: I think it was relevant [because] my employment case manager can sense the social [challenges] well, but the medical [challenges] have been important to have the physicians emphasize.

(Ahmad, T3)

At time point four, three participants had been granted disability pension, two were awaiting a final decision on their disability pension, and two were still undergoing a work capacity assessment that they had been instructed to complete in order to reach the decision-making stage. The network meetings thus seemed to facilitate progression towards final work capacity assessments.

Most participants described living alone and found social interactions stressful. For example, they said that internships, the process of the work capacity assessment, public transportation, and grocery shopping were difficult. The participants preferred to stay in the safe space of their own homes or take walks in secluded areas.

During and after the intervention, the participants described support needs. Several individuals had a long-term appointed support person to manage practical issues, such as finding a new apartment, attending doctors’ appointments, and applying for financial aid. Etibar explained his relationship with the support person:

The support person has planned that we will meet twice a month and we will talk about what is going to happen, or what [challenges] have arisen. Then I can talk to her about what I need help with, and about what is new.

(Etibar, T4)

The support persons were sometimes family members, but predominantly someone appointed by the municipality.

**Fig. 4:**
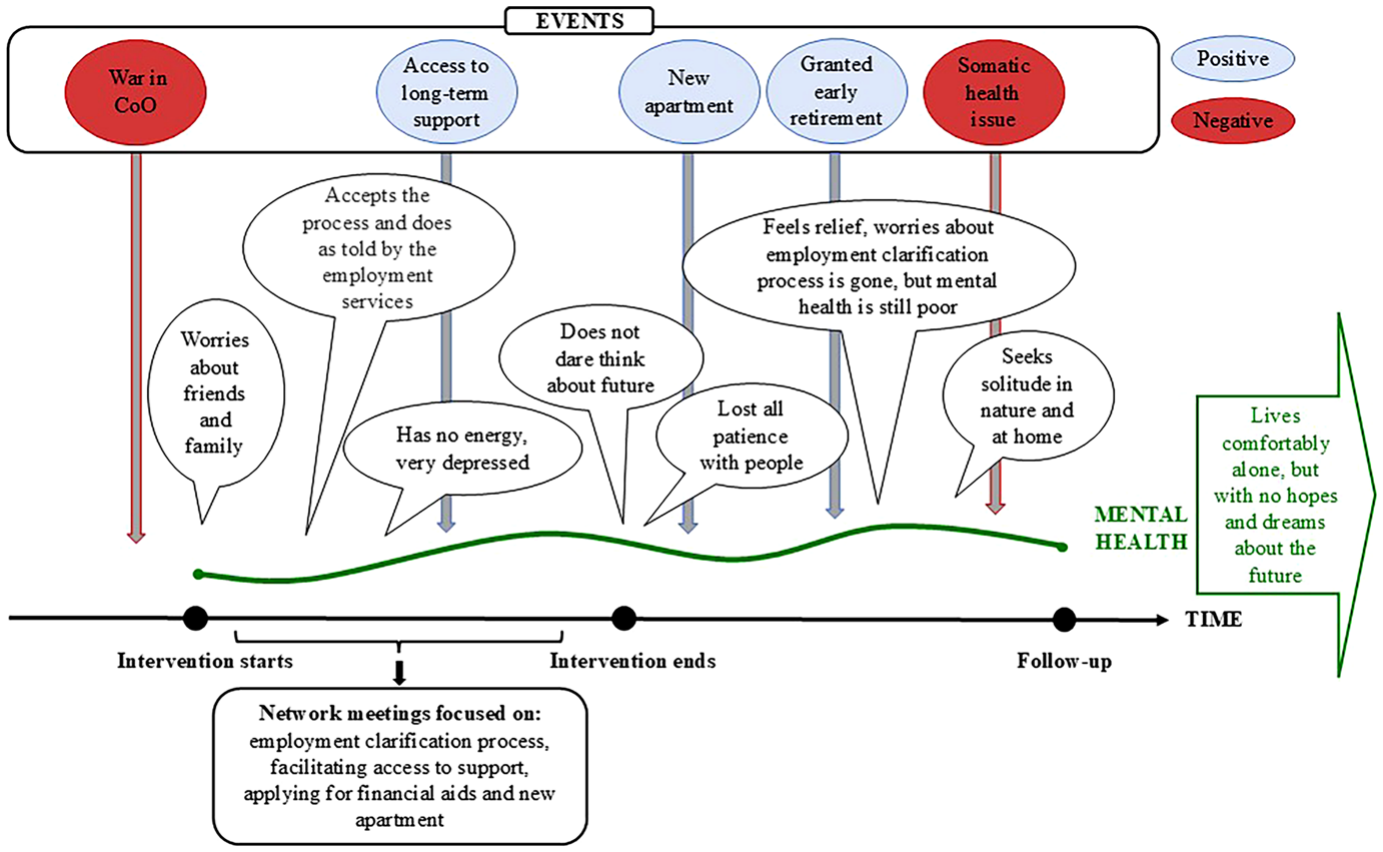
Example of mental health journey through time for pattern 3. *Co O:* country of origin

The support persons were especially important when challenging life events occurred. The participants explained that these life events were hard to deal with on their own and described how support persons helped them through them. During data collection, each participant encountered one or two challenging life events, often related to health issues. Due to their access to support persons, none of the participants described being left with an unmet support need at time point four. An example of the mental health journey for participants sharing pattern 3 is presented in Figure 4.

## Discussion

This study supports a social determinants perspective on refugee mental health, where emotional distress, post-migration stressors, and social aspects are closely intertwined (Hynie, 2018). We identified three distinct patterns of mental health change that differed regarding the need for support, encounters with challenging life events, and sense of agency. These patterns concur with the findings by Opaas et al. (2020), who point out substantial variation in how specialized mental health treatment influences individual refugee mental health over time. It supports the notion that specialized mental healthcare does not have a one-size-fits-all solution. Instead, mental healthcare must be personalized and agile enough to respond to the challenging life circumstances that can negatively influence treatments addressing emotional regulation (Gryesten et al., 2024). Individual tailoring thus seems the rule rather than the exception.

Within the intervention period, two of the three patterns saw positive mental health changes through multidisciplinary PTSD treatment and cross-sector collaborations to reduce post-migration stressors. This does not confirm causality between the intervention and positive mental health change in refugees with PTSD. However, it does emphasize the potential of the intervention to simultaneously address post-migration stressors and emotional distress, as recommended by the research and practice field of refugee mental healthcare (Miller & Rasmussen, 2024).

The mental health among participants in pattern 3 remained persistently poor over time, indicating that they did not benefit from the multidisciplinary treatment. Instead, they benefited predominantly from managing post-migration stressors, particularly because of access facilitated to long-term support and assistance with work capacity assessments. Reaching final assessment decision-making is one of the primary reasons refugees with PTSD engage in cross-sector collaborations (Attardo, Bruhn, Skovdal, et al., 2025). Specific to participants in pattern 3 was also that they felt too sick to be helped, and less hopeful about their futures. These feelings can reflect life situations where the number of different struggles, including mental, somatic, and post-migration struggles, make it challenging to engage in treatment. Value-Based Counseling to facilitate hope and meaning-making (Orang et al., 2023) alongside managing post-migration stressors might be more suitable than the multidisciplinary treatment provided in the intervention.

Participants following pattern 3 also described a preference for solitude and spending time in nature. We recognize that it can reflect severe impairment, which makes social interaction strenuous, or avoidance, which is a core symptom relevant to address in psychotherapy. However, exploring collaborations to support spatial relationships as an add-on to multidisciplinary mental health treatment might be relevant, as research hints that nature-based therapy can support mental health (Uldall et al., 2022).

Lack of social support was significant to the mental health journey of pattern 2. There were mental health improvements, but also a decline after the intervention terminated, which highlights the importance of long-term evaluations of interventions. Additionally, the decline suggests that for a subgroup of refugees, the loss of support can impede the lasting benefits of multidisciplinary treatment and collaborations to address post-migration stressors. Consequently, we agree with the recommendation made by Costa et al. (2025) to integrate collaborations with non-clinical services and lay persons in formal healthcare systems to adequately address the structural injustices shaped by social determinants for refugees, such as access to support. Including family members in treatment collaborations is another strategy that can promote long-term support for refugees and simultaneously address stigma among caregivers.

Participants in pattern 3 had access to comparatively more long-term support, often arranged during the intervention. It might explain why they did not describe unmet support needs or declining mental health at time point four. In a study of refugees resettled in Germany, Heidinger (2023) also argued that low human capital, driven by low language proficiency, makes it more urgent for refugee populations to access social support. Hence, formally linking the intervention to non-clinical, community-based, and voluntary services is a pivotal next step.

Across patterns, the participants encountered challenging life events, but they were more frequent in patterns 2 and 3, and often related to somatic health crises. This supports existing findings about the association between cumulative stressful life events, including somatic health events, and mental distress (Tibubos et al., 2021). This can partly explain why participants following patterns 2 and 3 did not consistently experience improvements in their mental health. Additionally, participants following pattern 1 felt a greater sense of agency and found that they could better cope with challenging life events. This concurs with the suggestion that it might not be the event itself that causes emotional distress, but instead the individual reaction to events and perceived capacity for action that is important (Schock et al., 2016). The potential mental health impacts of cross-sector interventions might thus be undermined when too many challenging life events occur without the perceived capacity for action or adequate support to address them.

Research has demonstrated that mental health social workers can act as formal system navigators, locating and negotiating access to services, addressing the various health and non-healthcare needs of refugees (Attardo, Bruhn, Audulv, et al., 2025). Working closely with such navigators and also general practitioners to mitigate the persistent influence of challenging events on mental health, especially health crises, should be a priority in cross-sector collaborations for refugees who experience complex care needs.

There were some indications of gender differences across patterns, with women tending to benefit more from the combination of multidisciplinary treatment and cross-sector collaborations addressing post-migration stressors than men, even though the numbers are too low to make firm conclusions. Other research has also found that particularly long-settled male refugees respond less well to mental health interventions compared to women, and they propose a broader mental healthcare approach that can address both mental, somatic, and practical problem (Opaas 2022). The intervention we explored did not address somatic problems directly, however, doing so could be key to providing the proper care for long-settled refugee men. Further research into gender differences in refugee mental healthcare populations is warranted to determine what drives gender differences.

Globally, there is a need for specialized mental healthcare advancements, which makes it relevant to explore whether the intervention is relevant to settings other than high-income countries. Research among internally displaced persons in Ethiopia residing in temporary shelters highlights that multi-sector psychosocial services are needed to address emotional distress and stressors such as unemployment concurrently (Gudeta & Seyeneh, 2025). Therefore, the relevance of the cross-sector PTSD intervention might extend beyond the context of high-income countries to include low-income and humanitarian settings as well. In fact, Ndlovu and colleagues (2024) argue the relevance of multi-sector psychosocial interventions in humanitarian crisis settings but find that few such interventions have been tested. While we acknowledge that the sectoral context in the present study differs from those in humanitarian crisis settings, the idea of utilizing cross-sector network meetings to integrate multiple services and expertise across sectors may be relevant.

### Limitations

This study offers insights into the mental health changes experienced by refugees and the factors that contribute to these changes. The application of the qualitative longitudinal approach facilitated an in-depth exploration of possible mechanisms central to changes in the mental health of refugees. QLR is, however, sensitive to dropouts, and not all participants could be reached at all timepoints. One possible concern with the POLA approach is the risk of having too little data in one pattern, which makes it challenging to provide a trustworthy description of the pattern (Kneck & Audulv, 2019). Despite the small sample size in this study, all patterns seemed robust, with at least five individuals represented in each pattern. Nevertheless, some factors could not be extensively explored and lead to robust conclusions due to the sample size, e.g., gender differences. There were differences in the proportions of men and women across the three patterns, but there was no data that suggested explanations for gender-related differences. Further research about gender-driven differences in specialized refugee mental healthcare is needed to inform intervention adaptation and address this limitation.

Our sampling strategy contributed to variation in the sample, since different life circumstances were intentionally selected. However, participants were mainly long-settled refugees who had lived in Denmark for an average of 20 years, as patients referred to the clinic typically have lived in Denmark for 15 years or longer prior to referral. Future studies could address this limitation by purposefully sampling recently arrived refugees. We recognize that our findings would have been different, and vocational rehabilitation perhaps more realistic, if participants had arrived in Denmark within recent years. Additionally, transferability of the results must also be considered, bearing in mind that only unemployed refugees from one outpatient clinic in Denmark were recruited. Future research could explore the professionals’ perspectives, as their intervention experiences are also relevant when considering adaptations to the cross-sector PTSD intervention to better help refugees who seek specialized PTSD treatment.

We acknowledge the potential for social desirability bias, which may have influenced the participants’ responses during interviews, as they may have been reluctant to disappoint the researchers or critique the intervention. To mitigate such risk, the participants were repeatedly informed that we were interested in their experience and how the intervention could be adapted to better suit their needs. Further, the interviewer was not involved in providing the intervention and emphasized this position to the participants at each time point.

## Conclusion

This study provided a longitudinal analysis of mental health changes over time for refugees who participated in a cross-sector PTSD intervention and identified three patterns. Despite the sample size, the study demonstrates that the cross-sector PTSD intervention does hold distinct mental health value that warrants sustained interest in cross-sector collaborations as an important approach to mental healthcare. Expanding the cross-sector collaboration to include multiple healthcare and non-healthcare organizations is a pivotal next step to advancing specialized mental healthcare for refugees.

## Supplementary Information

Below is the link to the electronic supplementary material.

Supplementary file1 (DOCX 25 kb)

## Data Availability

The data supporting the findings of this study are not openly available due to reasons of sensitivity and to preserve individuals’ privacy under the European General Data Protection Regulation.
