# Supplementary material for: Changes to Refugee Mental Health During and After a Cross-Sector PTSD Intervention: A Qualitative Longitudinal Study About the Influence of Social Support, Life Events, and Agency
Source: Cult Med Psychiatry. 2025 Oct 5;49(4):1265–85. doi: 10.1007/s11013-025-09944-1 (PMC12745305; doi:10.1007/s11013-025-09944-1)
Supplement: Supplementary file 1 — Supplementary file1 (DOCX 25 kb) [file 11013_2025_9944_MOESM1_ESM.docx]

**Supplementary file: Interview guides for two in-depth interviews**

Introduce the purpose of the interview and the three themes.
Ensure continued consent to participate in the study and consent to record the interview. Inform about anonymity, confidentiality, data storage, use of the data material in articles, and other dissemination.
Inform about the right to delete data and check if the interview has questions to ask.

| **End-of-intervention interview**  **(n = 22)** | **Follow-up interview**  **(n = 18)** |
| --- | --- |
| **TOPIC 1: THE INTERVENTION**   - How has it been to participate in the intervention and network meetings? - When looking back at the intervention, what has been the most important factor in how you feel now? - Can you describe your current collaboration with your employment case worker? Has it changed? - Aside from your employment case worker and the mental health professionals, are there other persons you would have liked to participate in the network meetings? | **TOPIC 1: LIFE AFTER THE INTERVENTION**   - What has happened in your life since the intervention and your treatment ended? - What about [individual agreements made at the third network meeting]? How did that work out? - What/who has supported, hindered, or created uncertainties? |
| **TOPIC 2: MENTAL HEALTH AND CURRENT LIFE**   - Can you describe how you are generally doing at the moment? - How does it compare to before starting treatment at the CTP? - Looking back, what do you think has been the most important for how you are currently feeling? | **TOPIC 2: MENTAL HEALTH AND CURRENT LIFE**   - Can you describe how you are generally doing at the moment? - What is your everyday life like? - What do you think has had the most significant influence on how you are currently doing? - How does it compare to before starting treatment at the CTP? How does it compare to when you finished treatment at the CTP? Can we try to draw it together on a piece of paper? |
| **TOPIC 3: THE FUTURE**   - What does the future look like to you? Expectations/worries/hopes/support needs? | **TOPIC 3: THE FUTURE**   - What does the future look like to you? Expectations/worries/hopes/support needs? |

**ROUNDING OFF THE INTERVIEW**

| Thank the interviewee for allowing me to follow their life and journey through the intervention period.  Explain plans for the follow-up interview and verify the contact information.  Explore if the interviewee has any questions. Thank you very much for your participation. | Thank the interviewee for allowing me to follow their journey through the intervention and afterwards  Explain the plans for analysis and explore whether the interviewee is willing to participate in further interviews in the future, and verify the contact information.  Explore if the interviewee has any questions. Thank you very much for your participation. |
| --- | --- |
